# Piranha-etched titanium nanostructure reduces biofilm formation in vitro

**DOI:** 10.1007/s00784-023-05235-4

**Published:** 2023-08-31

**Authors:** Khaled Mukaddam, Monika Astasov-Frauenhoffer, Elizaveta Fasler-Kan, Sabrina Ruggiero, Farah Alhawasli, Marcin Kisiel, Ernst Meyer, Jochen Köser, Michael M. Bornstein, Raphael S. Wagner, Sebastian Kühl

**Affiliations:** 1 Department of Oral Surgery, University Center for Dental Medicine Basel (UZB), University of Basel, Mattenstrasse 40, 4058 Basel, Switzerland; 2 Department Research, University Center for Dental Medicine Basel (UZB), University of Basel, Mattenstrasse 40, 4058 Basel, Switzerland; 3 Department of Pediatric Surgery, Children’s Hospital, Inselspital Bern, University of Bern and Department of Biomedical Research, University of Bern, Freiburgstrasse 15, 3010 Bern, Switzerland; 4 Department of Biomedicine University of Basel and University Hospital Basel, Hebelstrasse 20, 4031 Basel, Switzerland; 5 Department of Physics, University of Basel, Klingelbergstraße 82, 4056 Basel, Switzerland; 6 Institut für Chemie und Bioanalytik, Hochschule für Life Sciences, Hofackerstrasse 30, 4132 Muttenz, Switzerland; 7 Department of Oral Health & Medicine, University Center for Dental Medicine Basel (UZB), University of Basel, Mattenstrasse 40, 4058 Basel, Switzerland; 8 Institut Straumann AG, Peter-Merian-Weg 12, 4052 Basel, Switzerland

**Keywords:** Nanostructure, Antibacterial, Peri-implantitis, Surface, Piranha etching, Implant dentistry

## Abstract

**Objectives:**

Nano-modified surfaces for dental implants may improve gingival fibroblast adhesion and antibacterial characteristics through cell-surface interactions. The present study investigated how a nanocavity titanium surface impacts the viability and adhesion of human gingival fibroblasts (HGF-1) and compared its response to *Porphyromonas gingivalis* with those of marketed implant surfaces.

**Material and methods:**

Commercial titanium and zirconia disks, namely, sandblasted and acid-etched titanium (SLA), sandblasted and acid-etched zirconia (ZLA), polished titanium (PT) and polished zirconia (ZrP), and nanostructured disks (NTDs) were tested. Polished titanium disks were etched with a 1:1 combination of 98% H_2_SO_4_ and 30% H_2_O_2_ (piranha etching) for 5 h at room temperature to produce the NTDs. Atomic force microscopy was used to measure the surface topography, roughness, adhesion force, and work of adhesion. MTT assays and immunofluorescence staining were used to examine cell viability and adhesion after incubation of HGF-1 cells on the disk surfaces. After incubation with *P. gingivalis*, conventional culture, live/dead staining, and SEM were used to determine the antibacterial properties of NTD, SLA, ZLA, PT, and ZrP.

**Results:**

Etching created nanocavities with 10–20-nm edge-to-edge diameters. Chemical etching increased the average surface roughness and decreased the surface adherence, while polishing and flattening of ZrP increased adhesion. However, only the NTDs inhibited biofilm formation and bacterial adherence. The NTDs showed antibacterial effects and *P. gingivalis* vitality reductions. The HGF-1 cells demonstrated greater viability on the NTDs compared to the controls.

**Conclusion:**

Nanocavities with 10–20-nm edge-to-edge diameters on titanium disks hindered *P. gingivalis* adhesion and supported the adhesion of gingival fibroblasts when compared to the surfaces of currently marketed titanium or zirconia dental implants.

**Clinical relevance:**

This study prepared an effective antibacterial nanoporous surface, assessed its effects against oral pathogens, and demonstrated that surface characteristics on a nanoscale level influenced oral pathogens and gingival fibroblasts.

**Clinical trial registration**: not applicable

## Introduction

A frequent complication arising from dental implant therapy is the development of peri-implantitis, which has been defined as a plaque-associated pathological condition affecting the tissues around dental implants, and it is characterized by inflammation in the peri-implant mucosa and subsequent progressive loss of the supporting bone. Despite the different nonsurgical and surgical treatment strategies proposed, disease resolution and regeneration of the lost tissues is challenging, and even when achieved, recurrence may occur. In light of this limited predictability, prevention is of utmost importance. Therefore, the development of more sophisticated antibacterial implant surfaces that also allow for soft tissue integration is required [1, 2]. Nanoporous titanium surfaces have been shown to influence osteoblasts, inhibiting osteoclastogenesis and augmenting the release of osteogenic cytokines from both macrophages and osteoclasts [3]. Furthermore, nanoporous surfaces with pore diameters of approximately 74 nm have demonstrated enhanced induction of the HGF-1 response, which may provide additional benefits for soft tissue integration around the implant [4]. However, the literature describing the relationship between the nanoporous surfaces and HGF-1 cells and their antibacterial properties against oral pathogens is limited.

There are several factors that influence the quality of the peri-implant soft tissue seal, namely, fibroblast behavior and oral bacteria adhesion [5]. In addition, many other factors have been presented in the current literature, all concerning the physical-chemical properties of implant materials [6, 7]. Surface characteristics of dental implants or abutments, including roughness, hydrophilicity, and surface chemistry, are all significant determinants of the HGF-1 responses to the implant materials. The importance of the HGF-1 response is profound, and previous studies demonstrated that the proliferation of human gingival fibroblasts (HGFs) was significantly faster on smooth compared to rough abutment surfaces [8]. Therefore, cell-surface interactions of nanomodified surfaces have been proposed to assist in gingival fibroblast adhesion and support antibacterial properties through surface topographical alterations.

Titanium and zirconia implants are widely accepted as the implant materials of choice [9]. The transgingival part of dental implants can either be machined or polished, with previous studies suggesting that polished surfaces significantly enhance the integration of titanium abutments at the peri-implant tissue [10]. A change in the surface topography can be achieved through etching, a process utilized to alter the surface characteristics such as surface roughness. There are several types of etching, including sulfuric acid etching and piranha etching. However, the piranha solution, which is a mixture of sulfuric acid and hydrogen peroxide, has been shown to react with titanium to form a nanoporous surface. This effect resulted in an increased surface area of the transgingival part of the implant and modulated cell behavior [11].

Huang et al. [12] fabricated a novel titanium implant surface that enhanced osteoblast viability and presented inherent antibacterial activity through alterations to the surface topographical features. Surface analyses and biological evaluations indicated that a nanoporous surface may outperform current implant surfaces [12]. Hence, further analyses of this novel surface, its interactions with soft tissues, and its antibacterial properties are warranted. The aim of the present study was to determine how a titanium surface with nanocavities affects the viability and adhesion of HGF-1 and its properties against *P. gingivalis*. The hypothesis of the present study was that controlled nanostructured surfaces on commercially available polished titanium disks can be successfully created through piranha etching and might affect HGF-1 and *P. gingivalis*.

## Materials and methods

### Specimen production

To create the nanostructured specimens, NTDs of commercially available PT (Institute Straumann AG, Basel, Switzerland) (15 mm ×1,5 mm) were modified by first creating cavities measuring 1–2 μm through etching with H_2_SO_4_ 97%/HCl 36%/H_2_O (6:1:5) at the boiling temperature (approx. 140°C) for 1 min. After this, a second etching step was performed with H_2_SO_4_ 97%/H_2_O_2_ 30% (1:1) for 30 min at room temperature to create cavities with 10–20 nm edge-to-edge diameters on top of the first ones.

The surfaces of commercially available titanium (SLA and PT) and zirconia (ZLA and ZrP) were prepared as disks with diameters of 15 mm × 1.5 mm and used as controls. The PT surfaces were prepared by using dilute nitric acid to clean the surface, followed by washing in reverse osmosis purified water. The SLA surfaces were prepared by blasting the titanium with corundum particles, followed by etching with HCl/H_2_SO_4_. Similarly, the zirconia disks had either smooth, machined surfaces (ZrP) or microrough surface topographies (ZLA) that were produced with a mild large-grit sandblasting procedure followed by etching with hydrofluoric acid. All tested control groups were manufactured by Institute Straumann AG, Basel, Switzerland.

While PT and ZrP correspond to the clinically standardized polished and smooth parts often utilized for tissue-level dental implants, the SLA^®^ titanium (SLA) and ZLA^®^ zirconia disks (ZLA) represented the implants’ osseointegrated parts.

### Material characterization studies

#### AFM

Experiments were performed in a dry environment under a nitrogen gas atmosphere at room temperature. Five samples (NTD, SLA, ZLA, PT, ZrP) were examined by AFM with the intermediate and full contact modes. The topographical images and the average surface roughnesses (*R*_a_) were obtained with intermediate-contact mode AFM with a PPP-NC cantilever (Nanosensors™, Neuchatel, Switzerland). The sensor stiffness and frequency were *k*=28 N/m and *f*=160 kHz, respectively. Six different AFM images were obtained for every surface while successively reducing the image sizes from 20 μm^2^ down to 0.3 μm^2^. Due to the substantial amounts of data, only some AFM images are shown in the main manuscript. The adhesion force and work of adhesion measurements were performed with contact mode AFM using a PPP-CONT cantilever, with a sensor stiffness and frequency of *k*=0.1 N/m and *f*=11 kHz, respectively. To obtain the adhesion values, 30 force-distance curves were acquired for every surface.

##### Contact angle measurements

Prior to performing the contact angle measurements, the samples were cleaned by sequential ultrasonication in acetone, cyclohexane, and acetone. Water drop contact angles were determined within 10 min after solvent cleaning, and a DSA 10 Mk2 drop-shape analysis system (Krüss™, Hamburg, Germany) was used with the sessile drop method.

##### Antimicrobial effects of the surfaces on P. gingivalis

*Porphyromonas gingivalis* (ATCC 33277) was grown in thioglycolate (Biomerieux SA™, Geneva, Switzerland), enriched with 5 μg/mL hemin (Fluka™, Buchs, Switzerland) and 0.5 μg/mL menadione (VWR International™, Dietikon, Switzerland), and incubated anaerobically at 37 °C for 96 h. Bacteria were harvested in the stationary phase by centrifugation (8000 rpm, 5 min, RT) and resuspended in simulated body fluid [13] enriched with 0.2% glucose. Thereafter, the bacteria were allowed to adhere to the disks for 6 h at 37°C under anaerobic conditions to mimic initial adhesion. Following this, the adhesion and possible antibacterial effect were evaluated by:

*a)Conventional culturing.* The disks were dipped in 0.9% NaCl to remove the loose cells, and the back sides of the disks was decontaminated for 30 s with 70% ethanol. Thereafter, the disks were placed in 5 mL of 0.9% NaCl and vortexed at 2500 rpm for 1 min, followed by ultrasonication for 10 s at 3 W (Vibracell™, Sonics & Materials, Newtown, CT). One hundred microliters of the bacterial solutions were spread on Columbia® blood agar plates (BBL^TM^, BD Becton Dickinson; Allschwil, Switzerland). The plates were incubated for 7 days at 37°C under anaerobic conditions before the colonies were counted. The assay was repeated three times with triplicate samples (*n* = 9).

*b)Live/Dead* BacLight® Bacterial Viability Kit (ThermoFisher Scientific™, Basel, Switzerland) according to the manufacturer’s instructions. Samples were analyzed by confocal scanning laser microscopy (Leica SP8®, Leica Microsystems™ (Schweiz) AG, Heerbrugg, Switzerland) using a 63× oil objective (NA 1.40, HP PL APO CS2). The experiment was repeated three times with triplicate samples (*n* = 9). The volumes of the stained biofilms were measured with Imaris® (Imaris version 9.1; Oxford Instruments™; https://imaris.oxinst.com) for both labels used. The viability % was calculated as follows:

$$\mathrm{Viability}\;\%\;=\;100\times(({\mathrm{volume}}_{\mathrm{alive}\;\mathrm{cells}}/{\mathrm{volume}}_{\mathrm{total}\;\mathrm{cells}})/{\mathrm{volume}}_{\mathrm{totalcells}})$$

*c)Scanning electron microscopy.* The samples were dehydrated with stepwise increased concentrations of ethanol, dried at the critical point, coated with 10 nm of gold, and analyzed with 5000× magnification (SEM, Fei Nova NanoSEM 230®). The experiment was repeated twice with single samples (*n* = 2).

##### Cell culture

HGF-1 cells were purchased from ATCC (CRL-2014) and grown in Dulbecco’s minimal essential medium (DMEM) supplemented with 10% fetal calf serum (FCS) and 1% penicillin-streptomycin solution at 37 °C, 5% CO_2_, and 100% humidity, according to the corresponding tissue culture collection protocol. For all experiments, HGFs were used during passages 3–8. FCS, DMEM, and trypsin EDTA solution were obtained from Bioconcept™ (Allschwil, Switzerland). All other chemicals employed in this study were from Merck™ (Buchs, Switzerland) and of the highest purity grade. All cell culture experiments were performed using TPP® (Trasalingen™, Switzerland) plasticware.

##### Cell viability by MTT assay

To evaluate the effects of the various surfaces on the gingival fibroblasts, an MTT cell viability assay was performed. HGF-1 cells (30,000) were cultured on various disks in 24-well plates for 72 h, followed by the addition of thiazolyl blue tetrazolium bromide (MTT) at a concentration of 0.1 mg/mL. The HGF-1 cells were consequently incubated for an additional 4 h, and the reaction was finally stopped by adding 125 μL of dimethyl sulfoxide (DMSO). The MTT and DMSO were purchased from Merck™ (Buchs, Switzerland). All supernatants were harvested, and the optical densities were measured at 590 nm, as previously described [14]. Three independent experiments were performed in triplicate.

##### Immunofluorescence microscopy

The expression of actin and vinculin in HGF-1 cells post-incubation on the disks was also analyzed by immunofluorescence microscopy. HGF-1 cells (20,000) were cultured on disks for 72 h, fixed with ice-cold MeOH/acetone (1:1) for 15 min, and washed with PBS and stained with rabbit anti-human actin or mouse anti-human vinculin antibodies as previously described [15]. Alexa Fluor 647 goat anti-rabbit or Alexa Fluor 488 goat anti-mouse antibodies were used as secondary antibodies in these experiments. The nuclei were stained with DAPI. The images were collected and analyzed with an Olympus™ BX-51 microscope using a 40× objective and proprietary software. Three independent experiments were performed.

### Statistical analyses

The results for antimicrobial effects quantified by conventional culturing were evaluated to determine normality with the Shapiro-Wilk test. Thereafter, the results of the NTD group were compared to those of the other four control surfaces by Student’s *t*-test. The significance level was set to *p* < 0.05 (GraphPad Prism Version 9.4.1 for Mac, GraphPad Software, San Diego, California USA, http://www.graphpad.com). Cell culture experiments were performed at least three times, and statistical analyses were conducted with a two-tailed Student’s *t*-test. The statistical probabilities (*p*) were expressed as * for *p*<0.05, ** for *p*<0.01, and *** for *p*<0.001.

## Results

### Material characteristics

#### Topography

**Fig. 1:**
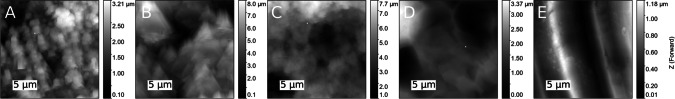
AFM topographical images (scan frame area: 20 μm × 20 μm); **A** NTD, **B** SLA, **C** ZLA, **D** PT, **E** ZrP. The scale bar indicates 5 μm

Figure 1 shows the AFM topographical (20 μm × 20 μm) images of the five studied surfaces. The applied piranha etching process resulted in an irregular nanostructured surface for NTD (A) and created cavities with 10–20 nm edge-to-edge diameters. SLA (B), which was produced by coarse grit-blasting with 0.25–0.5 mm corundum grit at 5 bar, followed by acid etching, had a high number of peaks/valleys across the surface.

The polished zirconia ZrP surface (e) showed the lowest average surface roughness. Due to the mechanical polishing, the characteristic elongated trenches were visible on those surfaces. No similar trenches were observed on the polished/pickled titanium or zirconia surfaces (d–e). Instead, the etching process of the NTD (A) surface resulted in the formation of nanometer-size cavities, whereas sandblasting of the SLA (B) and ZLA (C) surfaces led to the creation of hills/valleys across the surface. Trenches measuring 100 nm in length accompanied by cavities of similar size were observed for the ZLA surface (C). The machined PT (D) and ZrP (E) surfaces showed the most homogeneous topographies. Due to the polishing process, single micrometer grains were formed on the PT surface, whereas the mechanical treatment of ZrP resulted in the formation of several μm-long trenches.

#### Roughness

**Fig. 2:**
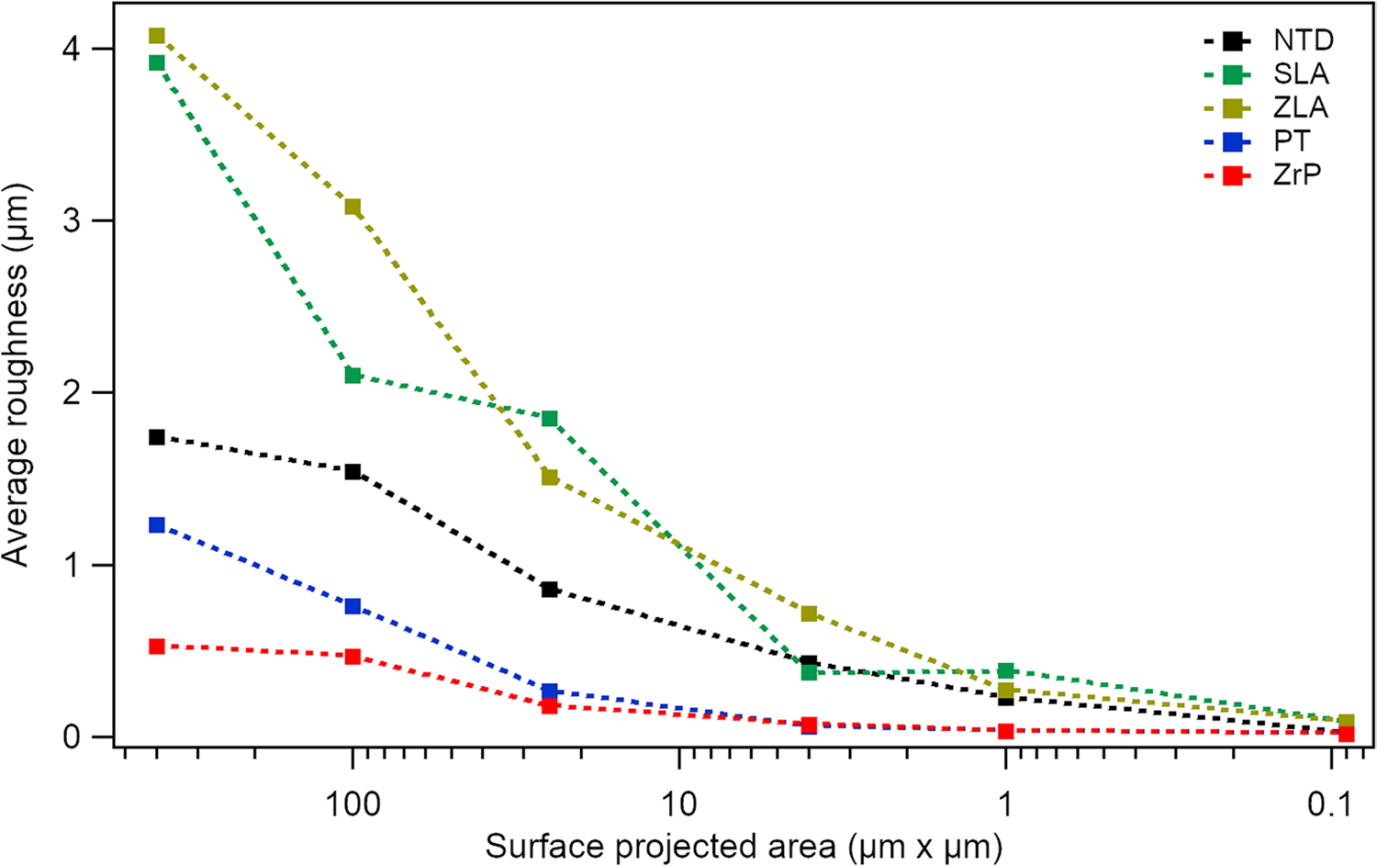
Average surface roughness parameters (*R*_a_) for NTD, SLA, ZLA, PT, ZrP

Figure 2 shows the extracted average surface roughness parameters (*R*_a_) for the differing AFM scan frame sizes. The six AFM images were recorded for each surface (data not shown here), and average roughness was extracted for every image. The image size was successively reduced for every surface. The scan frame areas were 20 μm^2^, 10 μm^2^, 5 μm^2^, 2 μm^2^, 1 μm^2^, and 0.3 μm^2^. The average roughness depended on the image size, and the main differences in the average roughnesses were seen with large-area scans. The polished ZrP showed the least roughness, whereas chemical etching of the SLA and ZLA resulted in large roughnesses. The nanostructured NTD surface showed a moderate average roughness. ZrP and PT showed the lowest average roughnesses, whereas ZLA and SLA exhibited very rough surfaces.

#### Adhesion force and work of adhesion

**Fig. 3.**
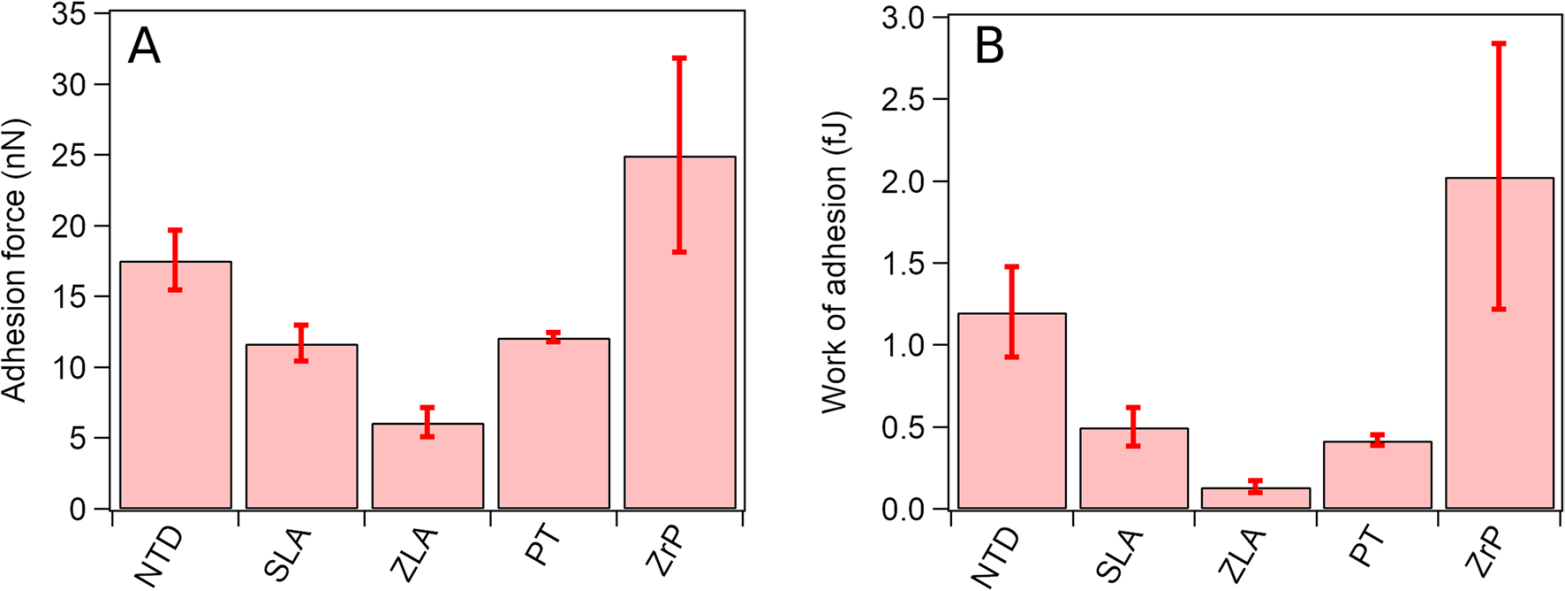
**A** Adhesion forces (nN) and **B** work of adhesion (fJ) for the five different surfaces

Figure 3 presents a comparison of the mean adhesion forces and mean work of adhesion measured for the different samples with silicon AFM tips. The data were acquired with contact AFM measurements of 30 force-distance curves for every surface. The adhesion forces and work levels ranged between 5–30 nN and 0.2–3 fJ, respectively. These measurements revealed that adhesion was inversely correlated with the average surface roughnesses. The flat, polished surfaces showed the largest adhesion, whereas the adhesion was lower for the rough surfaces.

ZrP exhibited the largest adhesion forces. Mechanical polishing of these surfaces resulted in reduced roughness (Fig. 2), and thus, the effective contact area between the AFM tip and the surface was increased. Consequently, the increased contact area on the flat surface led to increased adhesion. Accordingly, the high roughness reported for the ZLA surface led to a small effective contact area and thus the smallest adhesion force and work of adhesion. The adhesion forces of the NTDs were equal to 17.5 ± 2.1 nN. Due to the moderate roughness of the NTD surfaces, the adhesion levels were between the large adhesion values reported for the flat-machined ZrP surface and the roughest ZLA surface. While adhesion on the titanium PT surface might also be linked to the surface roughness, slightly differing behavior was observed for the titanium SLA surface. Despite the large roughness of the SLA surface, the measured adhesion forces and work showed slight increases, suggesting additional mechanisms for the adhesion forces.

#### Contact angle measurements

**Fig. 4:**
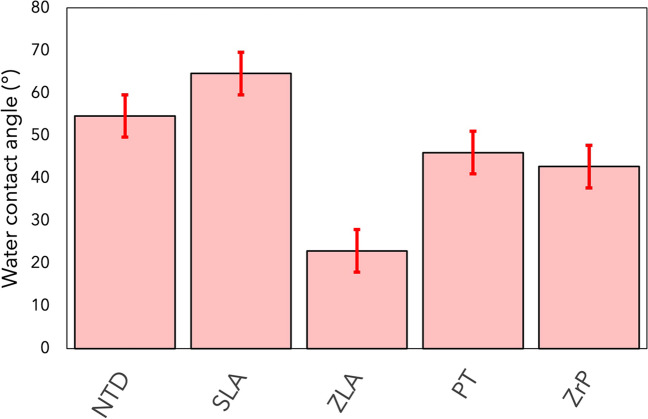
Water contact angles for NTD, SLA, ZLA, PT, ZrP. The error bars represent the standard deviations from at least three measurements per sample

It is well documented that enhanced hydrophilicities for titanium and zirconia surfaces enhance healing outcomes. All analyzed surfaces exhibited water contact angles well below 90°. Nanostructuration of the machined surface further increased its hydrophilicity, and all analyzed samples had contact angles well below that of SLA, which is considered the dental implant gold standard (Fig. 4).

#### Interactions between oral pathogens and specimens

**Fig. 5:**
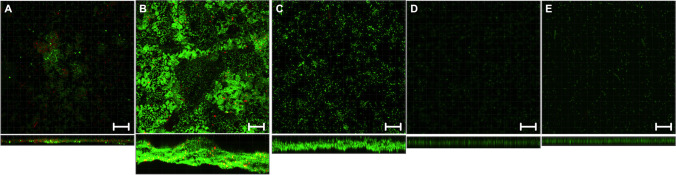
CLSM images of the five different surfaces (magnification 40×). **A** NTD, **B** SLA, **C** ZLA, **D** PT, and **E** ZrP. The scale bar indicates 20 μm

**Fig. 6:**
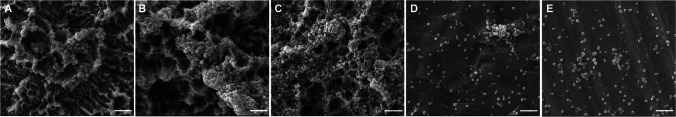
SEM images showing bacterial adhesion and formation of *P. gingivalis* biofilms (magnification 5000×), **A** NTD compared to marketed implant materials, **B** SLA, **C** ZLA, **D** PT, and **E** ZrP. The scale bars indicate 3 μm

Conventional culturing revealed a reduction in bacteria on the NTDs in comparison to the control SLA material with the rough surface (*p*<0.05). The comparisons with all other materials revealed no statistically significant differences (Table 1). Live/dead staining demonstrated the antimicrobial efficacy of the NTD (Fig. 5A) based on reductions in vital bacteria (Table 1). However, the range of viability was high within this sample group. No antimicrobial effect was observed for the control materials (Fig. 5B–E). Additionally, the nanostructured surface led to a statistically significant reduction in the biofilm thickness compared to those of the SLA (Fig. 5B) and ZLA (Fig. 5C and Table 1). SEM revealed additional bacteria on the rough titanium SLA (Fig. 6B) and ZLA (Fig. 6C) surfaces, followed by NTD (Fig. 6A), and the smooth surfaces of PT and ZrP (Fig. 6D, E). No differences were seen in the numbers of bacteria on the smooth marketed surfaces (PT and ZrP) and the NTD.

**Table 1:** Antibacterial effects of different materials evaluated by conventional culturing and viability staining. The significant difference (*p* < 0.05) between the NTD surface and the control group is indicated by *

Material	Conventional culturing	Live/dead staining	
	*CFU/mm*^*2*^	*Total volume (μm*^*3*^*)*	*Viability %*
NTD	5.27±0.8 × 10^4^*	7.60±0.16 × 10^4^	70.2 ± 20.3
SLA	1.53±0.75 × 10^5^	2.19±0.12 × 10^6^	95.3 ± 2.7
ZLA	6.16±4.86 × 10^4^	1.51±0.35 × 10^5^	96.1± 1.8
PT	2.25±0.84 × 10^4^	2.44 ±0.23 × 10^4^	96.2 ± 1.2
ZrP	6.63±5.77 × 10^3^	2.32 ±0.58 × 10^4^	95.4± 1.2

The cells were stained with a live/dead staining kit; live cells appeared green, and dead cells appeared red. The upper panels are top views of the adhesion of *P. gingivalis*, while the lower panels show cross-sections of the biofilms. Live/dead staining demonstrated the antimicrobial efficacy of NTD (Fig. 5A), revealing a reduction in vital bacteria.

The highest bacterial adhesion levels were found for the rough surfaces of titanium SLA and ZLA, followed by the NTD and the smooth surfaces of PT and ZrP. However, no differences were seen between the number of bacteria on the smooth marketed surfaces (PT and ZrP) and that of the NTD (Fig. 6).

### Interactions between human gingival fibroblasts and specimens

#### MTT assay

**Fig. 7:**
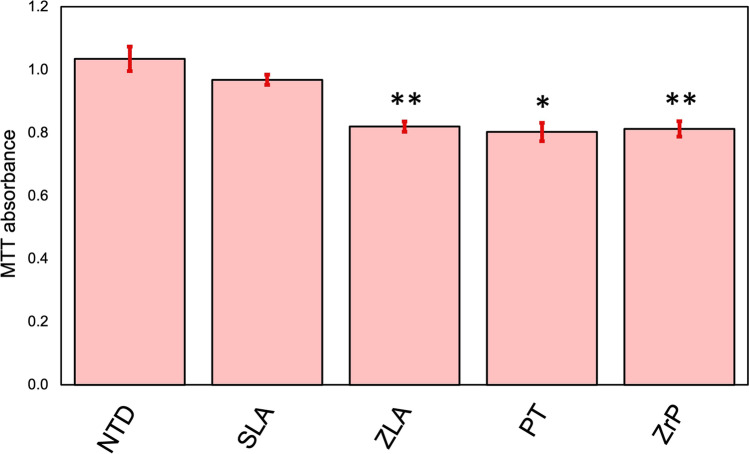
Comparison of the optical densities (ODs) for the indicated disks in the MTT assay

In the MTT assay, HGF-1 cells were incubated for 72 h on various disks. The HGF-1 cells cultured on the NTD disks showed the highest proliferation rates, followed by those on the SLA disks (Fig. 7), compared to the other specimens. In contrast, the HGF-1 cells on the ZLA, PT, and ZrP disks showed very similar absorbance peaks with intensities approximately 20% less than those of the NTD disks (Fig. 7). The experiments were performed three times in triplicate and the values represent the mean ± SD. *p<0.05; **p<0.01

#### Expression of actin and vinculin determined by immunofluorescence microscopy

**Fig. 8:**
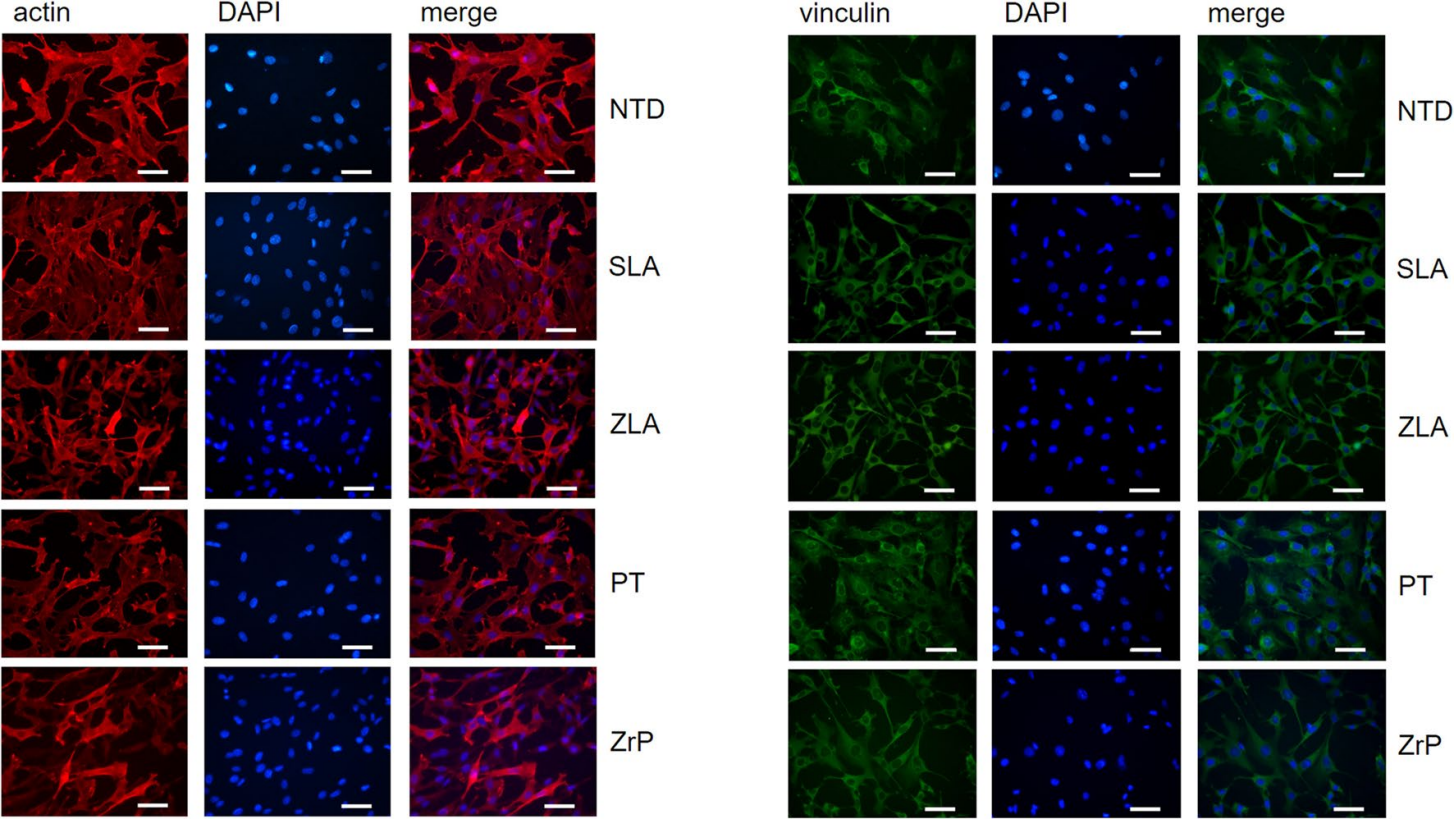
Immunofluorescence staining of actin and vinculin in HGF-1 cells upon cultivation on five different disks (magnification 40×). The scale bars indicate 50 μm

Immunofluorescence studies allowed analyses of the expression of actin (Fig. 8 left panels) and vinculin (Fig. 8 right panels) proteins in HGF-1 cells after 72 h incubation on various disks. All disks revealed very similar results. No differences were observed in the expression patterns for HGF-1 cells incubated on disks. The HGF-1 cells showed strong expression of actin (left panel) and vinculin (right panel), and they showed flat morphologies and appeared to adhere to the surfaces (Fig. 8).

## Discussion

The current study demonstrates that nanostructured surfaces can be successfully created via piranha etching of commercially available polished titanium disks. The etching process created cavities with 10–20 nm edge-to-edge diameters, and the polished ZrP material yielded the lowest surface roughness. The etched ZLA surface showed a low adhesion force and work of adhesion, with values of 5 nN and 0.2 fJ, respectively. Chemical etching of the SLA and ZLA disks produced the greatest surface roughnesses. The AFM topographical images and adhesion force measurements showed that the surface roughnesses were inversely correlated with the adhesion forces of the different surfaces, and the flat ZrP surface demonstrated the largest work of adhesion of approximately 2±1 fJ. The corresponding adhesion force was 25±5 nN. These findings were consistent with the conclusion that increased contact area between the cantilever and the flat surface resulted in strong adhesion, and the rough surfaces showed weak adhesion. The findings support the claim of a mechanical origin for the adhesion forces. Thus, the adhesion force was due to the tip and sample surfaces held together by interlocking. The titanium SLA surface, despite being rough, showed slightly increased adhesion, which suggests a more complex origin for the adhesion force.

Additionally, fewer *P. gingivalis* bacteria were observed on the NTD compared to the SLA, and the SEM images also showed additional bacteria on the rough surfaces. It is well established that rough surfaces facilitate biofilm formation. Despite the fact that our findings supported this claim, the rule seems to be ductile. The NTD had a surface roughness of approximately 2 μm, while the SLA and ZLA disks were twice as rough, and the PT and ZrP showed average roughnesses of approximately 0–1 μm. In contrast to the surface roughness, the NTD seemed to hinder biofilm formation most effectively. Given that the vast majority of bacteria are surface-attached cells, it is crucial that the bacteria-surface interaction is understood to prevent peri-implantation.

The HGF-1 cells demonstrated the greatest proliferation rates on the NTD disks. However, the immunofluorescence studies showed no significant differences in actin and vinculin expression patterns when the HGF-1 cells were incubated on the five different disk surfaces.

Cell proliferation on the titanium or zirconia surface was largely influenced by the surface topography of the implant. Therefore, alterations to the surface roughness can have a knock-on effect on soft-tissue integration [16, 17]. Guo et al. [18] conducted a review of the literature to assess soft-tissue integration with titanium implants. Poor epithelial attachment and the absence of direct collagen-implant integration were responsible for inferior soft-tissue integration around dental abutments. It was also emphasized that several cellular functions must be established and modulated, including HGF-1 proliferation, to achieve early soft-tissue integration. Beyond this, novel implant surface designs must ensure that the presence of oral bacteria is minimized [18].

Shifting the focus from the influence of the dental implant surface topography on the rate of osseointegration to interaction of the transgingival part of the implant with the peri-implant soft tissue is a crucial aspect of understanding the reasons for implant failure and the pathogenesis of peri-implantitis. Petrini et al. [19] highlighted this fact and evaluated the responses of HGFs to titanium implant disks with different micro- and nanotopographies created through sandblasting and dual-etching. The results of this study were consistent with the present findings in that double-etched titanium surfaces exhibited significantly higher surface roughnesses. Moreover, the titanium disks with nano-micro and macro topographies showed increased proliferation of HGFs. Hence, these findings collectively support the notion that the nanoporous titanium surfaces promote the adhesion and proliferation of HGFs at the peri-implant site.

A 2018 study by Gulati et al. [20] described a nanopore surface modification created by electrochemical anodization that resulted in dual micro- and nanorough horizontally aligned titanium nanopores. It was also emphasized that the nanoporous surface reduced the proliferation of macrophages, augmented osteoblast and fibroblast activities, and stimulated the adhesion of osteoblasts and fibroblasts to the implant site [20]. Miao et al. [21] also recognized the potential benefits of nanocavity surfaces while focusing on the effects of this novel topography on MG-63 osteoblasts, human gingival epithelial cells (HGECs), and HGFs. Nanocavities, which were created through an alkali-hydrothermal treatment, demonstrated enhanced osteogenic activity and a notable improvement in the attachment of HGECs and HGFs, which is consistent with the present findings. Moreover, the results of this study warrant the conclusion that a nanoporous titanium surface may promote both bone and soft tissue healing, enhance soft tissue integration, and increase the success and survival of dental abutments [21].

All surfaces analyzed in the present study exhibited water contact angles well below 90°, with the incorporation of nanocavities on the titanium surface, further increasing the hydrophilicities of the materials. The surface wettabilities of dental implant materials determine the resulting cascade of events at the peri-implant site, and highly hydrophilic surfaces are more desirable than hydrophobic materials [22, 23]. Several factors influence the wettability of a material, and Rohr et al. [24] observed a decreased water wettability of zirconia following prolonged air storage. This factor must be considered when producing and storing nanostructured dental implants or abutments.

Although the evidence presented in the few studies that investigated nanoporous titanium surfaces was promising, the literature indicating how these nanocavity surfaces affect the viability and adhesion of HGF-1 is lacking. Mukaddam et al. [25] aimed to address this oversight by subjecting titanium disks to piranha etching and comparing to commercially available machined titanium disks. The findings demonstrated that the nanocavities reduced cell alignment along the machined structures but did not have an adverse effect on the proliferation of HGF-1 when compared to the control disks. Beyond HGF-1 viability and adhesion, this study also mirrored the findings of the present study regarding actin and vinculin expression patterns, and no differences were observed between the nanostructured disks and the machined disks following incubation. However, the earlier study did not show antibacterial effects against *P. gingivalis* with nanocavity sizes of 10–20 nm produced by piranha etching. In contrast, the present study showed a notable reduction in *P. gingivalis* bacteria on the NTD surface following incubation. While the current study used commercially available pickled titanium disks for piranha etching, the previous study by Mukaddam et al. [25] employed machined titanium disks, which might explain the different results. However, this reduction was validated by Astasov-Frauenhoffer et al. [26], who assessed bacterial colonization, specifically that of *P. gingivalis colonization*, on nanostructured titanium surfaces. A notable reduction in bacteria was observed for the nanostructured titanium compared to the rough-surfaced control materials following conventional culturing. However, this study did note that similar results were observed with smooth-surfaced control materials. Investigations into the antimicrobial efficacy of nanoporous titanium revealed reductions in the vital bacterial populations by up to 70% [26].

Given the abovementioned findings concerning the role of nanocavity diameters in the antibacterial properties of surfaces, Ferrá-Cañellas et al. [4] sought the optimal nanopore diameter to improve the HGF response and soft tissue integration. Their findings suggested that nanopores with diameters of approximately 74 nm offered the best HGF response with regard to soft tissue integration [4]. The literature comparing nanoporous titanium surfaces, however, is limited, with few studies investigating the antibacterial effects of nanostructured surfaces against oral pathogens such as *P. gingivalis.* Hence, future investigations must incorporate this outcome measure into their methodologies. Additionally, since *P. gingivalis* is not the first species to adhere to implant surfaces in vivo during biofilm formation, it is crucial that future investigations assess colonization with other species, such as *S. oralis*, and the development of multispecies biofilms.

## Conclusion

Although highly improved antimicrobial efficacies were not detected, the NTD surface did present results comparable to those of commercially available surfaces and could be considered an alternative approach for surface preparation.

## Data Availability

The datasets generated during and/or analyzed during the current study are available from the corresponding author on reasonable request.
